# Artificial topography changes the growth strategy of *Spartina alterniflora*, case study with wave exposure as a comparison

**DOI:** 10.1038/s41598-017-16122-w

**Published:** 2017-11-17

**Authors:** Hualong Hong, Minyue Dai, Haoliang Lu, Jingchun Liu, Jie Zhang, Chaoqi Chen, Kang Xia, Chongling Yan

**Affiliations:** 1 Key Laboratory of the Ministry of Education for Coastal and Wetland Ecosystems, Xiamen University, Xiamen, 361102 P.R. China; 2 State Key Laboratory of Marine Environmental Science, Xiamen University, Xiamen, 361102 P.R. China; 3 Key Laboratory of Urban Environment and Health, Institute of Urban Environment, Chinese Academy of Sciences, Xiamen, 361021 P.R. China; 4 Department of Crop and Soil Environmental Sciences, Virginia Tech, Blacksburg, VA 24061 USA

## Abstract

This paper reports findings about the growth of *Spartina alterniflora* (Loisel.) near an engineered coastal protection defences to discover the potential influences on vegetation growth from the artificial topography. Impacts of the artificial topography on the sediment element composition were detected by comparing the fixed effects caused by artificial topography and wave exposure using linear mixed models. Surficial sediments under the impacts of artificial topography contain elevated levels of biogenic elements and heavy metals, including C (and organic carbon), N, S, Al, Fe, Mn, Cu, Zn, As, Cd, Cr, Ni, and Pb. The results showed that element enrichment caused by artificial topography reduced the vegetation sexual reproduction. Contrary to the potential inhibition caused by direct wave exposure, which was due to the biomass accumulation limit, the inhibition caused by artificial topography was related to the transition of growth strategy. The contents of Cu, Mn, N, Ni, S and As in the sediments were critical in considering the relationship between the change in the sediment element composition and the alteration in the plant growth. Our study emphasizes the importance of rethinking the impacts of coastal development projects, especially regarding the heterogeneity of sediment element composition and its ecological consequences.

## Introduction

Located in a marine-terrestrial interface, salt marshes are an ideal location to explore the phenomenon of plant zonation. Coupled with different food sources^1^ and vegetative physiological impacts on the niche^2,3^, plant zonation provides a wide range of habitat for diversiform species. The homeostasis of other organisms, such as soil microbes^4^, would be broken as consequences of the disorder of plant zonation. Plant zonation is also one of the crucial factors in intertidal ecosystem functions, such as carbon dioxide fluxes^5^ and biogeochemical processes^6^, which dictates the value of coastal ecosystem services^7^. The complex of plant zonation is described as a comprehensive integration from a number of ecological factors^8^. As a subject drawing long-term attention, the causes of plant zonation at the intertidal zone are usually explained by a series of drivers varying from physical stresses such as flooding^9,10^, salinity^10,11^ and nutrient shortage^12^, to biological interactions such as competition for light^13^ or nutrients^14,15^, as well as top-down control by herbivores^16^.

The effect of sediment element composition on intertidal vegetation growth is important for plant evolution and persistence^17–19^. As an important resource for synthesis of amino acid^20^ and some other important metabolites^21^ in plants and influencing plant physiological function^22^, nitrogen is considered as a major nutrient that limits the primary production of coastal vegetation^23^. Nitrogen enrichment has a positive impact on intertidal vegetation growth by increasing biomass production^24^, leaf biomass ratio^25^ and salt tolerance^26^. Moreover, some mineral elements such as Cu, Ni and Mn are also involved in plant metabolism by forming active sites for enzymes (for example, superoxide dismutase^27^ for reactive oxygen species scavenging and cytochrome c oxidase^28^ for photosynthesis). The disparity of element content further alters both the floral nutritional strategy and relative competitive advantage^12,29^, which might lead to an increase of ecological diversity for various organisms^30^.

Resulting from the distinct spatial distribution of environmental factors in the intertidal zones, plant zonation has a harmonious balance after a long-term evolution of intertidal ecosystem. However, humans might begin a new movement in the evolution process at the coastal zone with the rapid development of the coastal engineering. Reclamation activity occurs all around the world^31,32^, and its history can be dated back to more than 1000 years ago^33^. China has an even longer reclamation history. The Hanhai seawall in Jiangsu Province was built in 767AD. The original purpose of reclamation was to prevent inundation of seawater. However, today, large and increasing areas of the intertidal zones are occupied under the pursuit of extra land. Wetlands are converted into land for agriculture or industry, and the natural species-rich habitats are lost during this process. Reclamation reduces the biomass of plants^34^ and threatens their survival, and the reclamation at some critical regions might even cause global consequences^35^. The environmental consequence of reclamation also includes interruption of natural sediment transportation^36^, slowdown of the tidal flow^37^, decrease of C and N sink^34,38^, and increase of heavy metal mobility^39^.

Contrary to the ability to evoke a large transition in the intertidal zones, the knowledge about the profound influences caused by coastal construction on the complex ecosystem is faltering and sometimes ignored^40^. Several studies have focused on the environmental consequences of coastal reclamation and seawater flooding, and the block of tidal exchange was mostly considered in these studies^34,39,41–49^. However, the change in sediment element composition caused by artificial topography and its ecological consequences still need to be revealed. There is also some evidence implying that element discrepancy and physical stress are the drivers underlying the natural intertidal zonation pattern^12^, but empirical research is too sparse to illuminate the potential impact of artificial topography.

The recent coastal development projects of China cause changes in the patterns of sedimentation^36^, and have a complex influence on the distribution of species^40,50^. This also highlights the importance of exploring the potential ecological consequences caused by artificial topography. The present article presents a case study on a semi-open abeyant seawall, where construction was halted, which would causes a semi-open topography finally leading to accumulation of biogenic and mineral substance. The purpose here is to: (i) monitor element composition alterations in the sediment as a response to artificial topography; (ii) reveal the transition of plant growth under the interference of artificial topography; and (iii) probe the possible mechanism actuating the vegetative response to element composition changes under the influence of artificial topography.

## Results

### Effects on the sediment element composition

**Figure 1:**
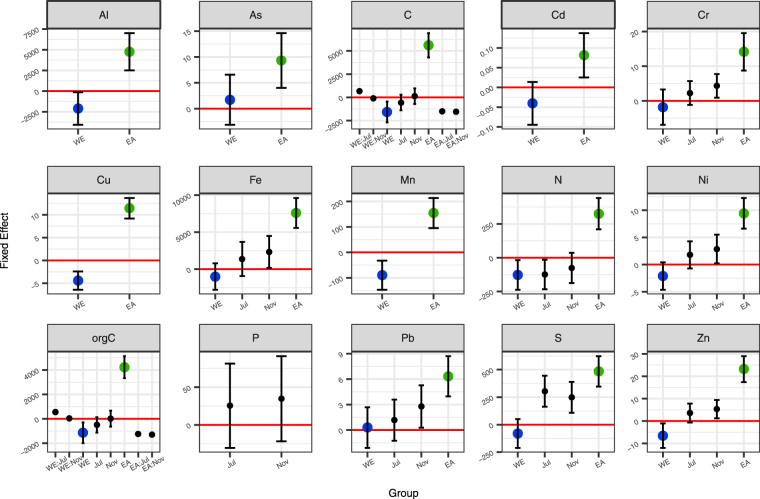
Fixed effects on the element composition of the sediments. Bars represent 95% confidence intervals. Blue represents direct wave exposure (WE) and green represents sites near the semi-open abeyant seawall (EA).

The effects of seasons and sites on the sediment element composition were extracted by linear mixed models (LMMs) (Fig. 1). The results of analysis of variance (ANOVA) and *post hoc* analyses can be found in Table S1. The contents of the elements, except P, were higher at the sites near the seawall (EA) compared to the control (CL), while there were no or slight differences between the sites under wave exposure (WE) and control (CL). Apart from the effects of the abeyant seawall, season was also a factor that had significant effects on the accumulation of elements such as S, N, Ni and Fe. The interaction between EA and season was also notable for C and organic carbon (orgC) (Table S1a,b). P contents in the sediment were stabilized across the sites and seasons.

### Effects on the vegetation growth

**Figure 2:**
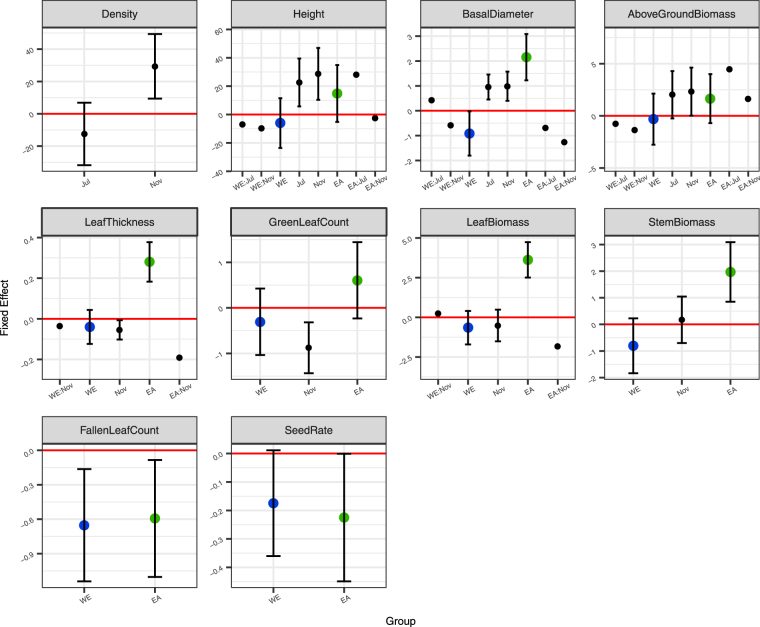
Fixed effects on the ramet growth traits of *Spartina alterniflora*. Bars represent 95% confidence intervals. Blue represents direct wave exposure (WE) and green represents sites near the abeyant seawall (EA).

We investigated the density as well as the mean height, basal diameter, and aboveground biomass per ramet through the growth of *S. alterniflora* (Fig. 2). The growth of *S. alterniflora* usually starts in March and ends in November at Rudong. The results of ANOVA and *post hoc* analyses are provided in Table S3. The height, basal diameter, and aboveground biomass were all influenced by artificial topography and season (Fig. 2). However, the effect of artificial topography on these three traits is enhanced in the middle of the growth season of *S. alterniflora* compared to the other two seasons (Table S4b–d). Season is the only control factor of the density, while the variation between sites might also have an impact manifested as random effects (Table S4a). We also included the traits relating to leaf development (i.e., the mean leaf thickness, green leaf count and leaf biomass per ramet) into the monitoring. Leaf thickness and leaf biomass were increased by EA, which is similar to the patterns of basal diameter. However, the *post hoc* results reveal that the effects of EA on these three traits were not significant at the end of growth season (Table S4c,e and g). Both seed setting and leaf falling were downward as responses to EA and WE. Although it needs further data in a wider region since the fluctuation of these two traits hampers the comparison between levels, the site-level comparing based on linear models still shows that EA caused the decline of seed setting rate (Table S7).

### Transition of the growth strategy

**Figure 3:**
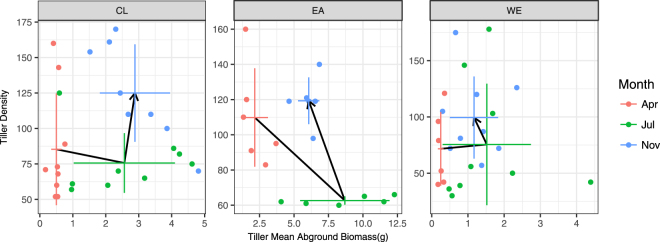
Seasonal switch of the trade-off between ramet density and mean ramet aboveground biomass. The horizontal and vertical bars represent the standard deviations and the black paths trace the development of traits throughout the growing season.

**Figure 4:**
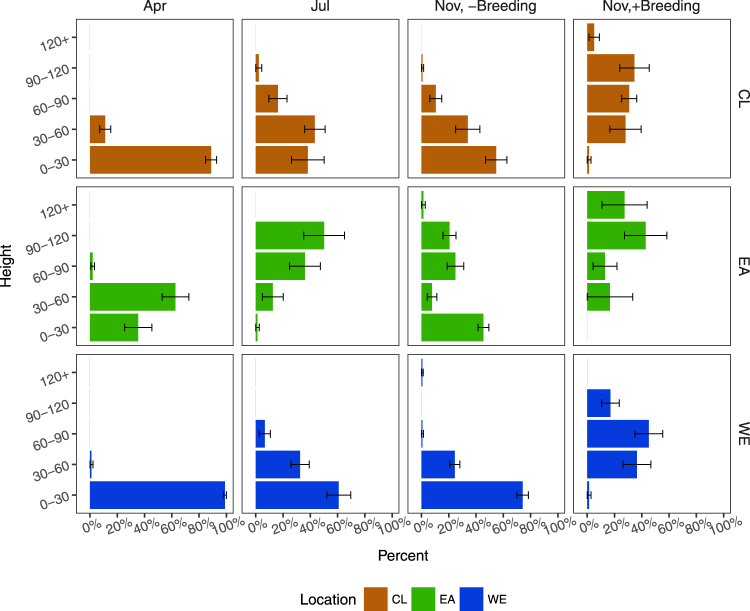
Comparison on the height structure of the *Spartina alterniflora* population between sites. Bars represent means ± standard errors.

Density-ramet growth trade-off is one of the key relationships that is used to understand plant energy use strategies. Here, we found that both wave exposure and element accumulation played key roles in this trade-off. As shown in Fig. 3, a similar development strategy (i.e., the common pattern of ramet biomass increased in the summer and the ramet density increased in the autumn) was found among different groups. However, the deviation of growth traits was different between groups (Table S4). In spring, the standard deviations of the ramet mean aboveground biomass at the CL and WE sites were lower than the standard deviations of the ramet density. In summer, the standard deviations of the ramet density at the EA sites were lower than the standard deviations at the CL or WE sites (Table S8). Note that compared to the control sites the potential reduction in the seed setting rate at the EA sites (Fig. 4) did not turn into an increase in the ramet density (considered as asexual reproduction).

The difference of the height structure among sites is shown in Fig. 4. Under the effects of EA, the height structure tended to be inverted compared to the CL and WE sites in summer. EA also increased the proportion of relative higher ramets in autumn, regardless of whether ramets underwent seed setting or not.

### Control factors on the ramet growth

**Figure 5:**
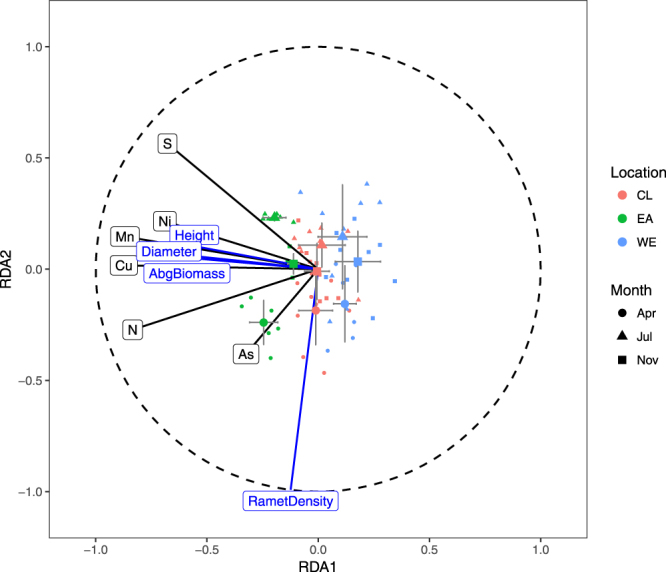
Visualization of cross-seasonal control factors of growth based on RDA results. The horizontal and vertical bars represent the standard errors of sample RDA loadings.

The RDA result is visualized in Fig. 5. Distribution of the twelve elements in the current study could explain 55.8% of the total variation in the growth traits of *S. alterniflora*, 55.4% of which could be explained in the first two RDA components. The RDA suggests that 6 elements, i.e., Cu, Mn, N, Ni, S and As, were critical in considering the relationship between the change in the sediment element composition and the alteration of the growth of *S. alterniflora*. The indicators more relevant to ramet growth, i.e., mean ramet height, mean ramet basal diameter and mean ramet aboveground biomass, were positively correlated with each other, while they were nearly fully independent of the ramet density, which is more related to the population growth. All of the six elements selected by RDA were positively related to the ramet growth, while only N and As were positively correlated to the ramet density and S seems to be a negative control factor in ramet asexual reproduction.

**Figure 6:**
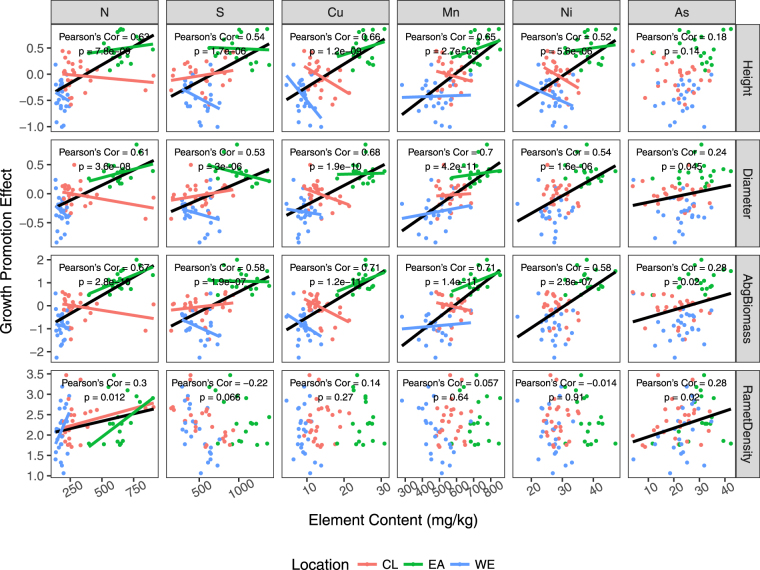
Scatterplot matrix of the correlation between growth traits and elements selected by the result of cross-seasonal RDA. Black (across groups) and colored (within groups) lines represent the significant correlation between growth traits and sediment element contents.

Robust linear models between growth traits and elements screened out by RDA (Fig. 6) reveal the detailed patterns of the alterations of growth traits, which are mostly in accord with the RDA result. One interesting aspect ignored by RDA is that, although the contents of elements were globally positively correlated to the ramet growth traits, some of them, including N, S, Cu, Mn, and Ni, showed different and even opposite trend within the group.

## Discussion

Waves provide the energy source to generate the physical and chemical gradients in coastal regions^51^. The existence of the semi-open abeyant seawall enhanced the accumulation of both the biogenic elements and heavy metals in the *S. alterniflora* wetland. Some previous studies on intertidal zone element distributions concluded that the colonization of *S. alterniflora* induced the accumulation of both organic matter^52–55^ and mineral elements^56,57^. Moreover, orgC and clay mineral might have accumulation trends along a time sequence of salt marsh development^58^. However, our results showed a greater accumulation of element concentrations (Table S3), which might be caused by the seawall, compared to a previous study in 2010 when the seawall did not exist^57^. As reinforcement to our demonstration, the relationship between element contents and distance from the seaward edge of vegetation (Fig. S3) clearly shows that the sediment element composition at the EA sites were deflected to the common spatial distribution trends at intertidal zones. This indicates that the existence of artificial topography has also promoted element accumulation. Under the effect of artificial topography, tidal dynamics were reduced and the accumulation of both clay minerals (indicated by increasing Al contents^59^) and plant litters (indicated by increasing orgC^60^) would be the reason for element enrichment (Fig. S2).

Although some studies have indicated that the pore water circulation regimen is more important than the sedimentary environment in the intertidal biogeochemical process^61^, our results emphasize that it is also important to take the sedimentary environment into account when considering the interactions between the land and the ocean. The present study, in accord with some previous research^62–64^, reveals that the change in the sedimentary and transport process has important roles in the distribution of the element contents at multiple scales and locations.

The eutrophication caused by the artificial topography increased the abundance of the ramets with an elevated height (Fig. 4) in the present research, which is consistent with some other results reported by peers^12^. The increase in the nutrient supply, especially N and Cu, might be the reason for the growth promotion. It needs to be noted that the gather of plant litter seems to be the only cause for N fertilization, while the Cu accumulation was caused by a synthetic effect including the plant litter and sediment transportation, as indicated by the result of multiple linear regressive analysis (Table S6). The growth-promoting effect of N on *S. alterniflora* is well established^65^. *S. alterniflora* is a plant with a relatively higher N demand. It is, therefore, not surprising that the increase in the nitrogen caused a more flourishing population. In the presenting study, the facilitating of metals, including Cu, Mn and Ni, has also come to light. The facilitating effects of metals on *S. alterniflora* should be due to the formation of enzyme active site, which needs the participation of correct metals. This is in accordance with a previous study on mangroves^66^, another phytogroup growing in the intertidal zones, showing that iron limits the seedling growth of five plant species living in the intertidal zone. This could be explained by the uptake of element during the vegetation growth and implies the possibility of element limit to the ramet growth. It is also possible that the structure of herbivores could shift the population structure of the vegetation^16^. However, in the presenting research, no distinct trace of this top-bottom control could be found. No evidence of gnawing was found during our field work, and the EA did not increase the number of withered leaves throughout the growing season (Fig. S7). In summary, the heterogeneity of the elements seems to be the dominant factor controlling the population structure at the EA sites in this research.

The presenting study emphasis that the complex interaction between halophyte growth and sediment element composition might be one of the key factors controlling the plant growth strategy. The sediment element composition is criticial for intertidal vegetation evolution and persistence with numerous of studies throwing light on this field^17–19^. The relation between the content of plant growth promoting elements and plant growth is a contradiction entity. On one aspect, the availability of these elements, relieves the stress of nutrition demand and also alleviates some physical stress by supplying the resource to form resistant material. Globally, the contents of these elements are positive related to the growth of plant^22^, especially at the intertidal zone under nutrient limitation. However, the impacts of these elements take place by assimilation into plant tissue and remove from sediments^67^. The result of this process, opposite to the global pattern, is that the increase of plant biomass is couple with decrease of plant growth promoting elements. This different correlation between group and global level emphasizes that it is the sediment element composition that mainly affects the vegetation growth, rather than that the plant uptake mainly influences the sediment element contents under the effect of artificial topography.

However, from the evidence revealed by the height structure and allometric growth, the promotion of ramet growth might not be a positive impact on the persistence of *S. alterniflora*. As a response to EA, the growth or development of the lower ramets might be under extreme inhibition, which could explain the reduction in the abundance of lower ramets throughout the growth season (Fig. 4). Moreover, EA increased the seed setting threshold for ramets, with the result that part of the relatively high ramets were kept in the vegetative growth stage as an effect of EA, whilst most parts of the ramets with a similar height proceeded to sexual reproduction under natural conditions (Fig. 4).

A higher intraspecies competition condition caused by EA by the way of increasing nutrient supply, might be the reason for this phenomenon. Eutrophication removed the limit on the nutrient demand, but the energy coming from solar radiation was still the controlling factor for population structure^13^. As an effect of EA, ramets had to grow higher to ensure that they received enough light for photosynthesis. Additionally, the allometric relationship between aboveground biomass and height (Fig. S8) shows that the biomass increased faster than the height following the increase of ramet height, and this might be due to the hollow-cylinder-like structure of the *S. alterniflora*, and the fact that basal diameter and height increased together (Fig. S8). Intraspecies competition for light leads to higher ramets, which might make it difficult for the *S. alterniflora* population to achieve enough energy to trigger sexual reproduction.

By controlling the energy use strategy and impacting both the expansion rate and competitive advantage, the population dynamics are a key factor for the survival of one species^68^. Both WE and EA impact the growth traits of *S. alterniflora* compared to the CL sites. A reduction in the seed setting rate was found at WE and EA sites (Fig. 2c), whereas WE reduced the mean ramet biomass whilst EA promoted the vegetative growth. The mechanism underlying this pattern seems to be different between WE and EA; the former might be controlled by whole plant growth, whereas the latter might be controlled by growth priorities since the ramet height and biomass at the EA sites was greater than the CL ones. Seasonal fluctuations in the density–ramet biomass relationship (Fig. 3) revealed that the vegetation development strategy shifted from ramet biomass to density fixing in summer at the EA sites.

Changes in asexual reproduction strategies were also involved in response to the integrated effect of waves. In spring, there was extreme variation in the ramet density with a similar average ramet aboveground biomass, which implied that the main controlling factor of the density-ramet biomass trade-off was density rather than ramet growth. However, the plant density remained the main controlling factor at the WE and CL sites, whereas at EA sites the population growth was controlled by ramet growth to some extent. At these sites, the density was decreased and fluctuated within a narrow band in summer. However, the aboveground biomass of the ramet exhibited a significant increase and fluctuated within a wide range, indicating that at this stage the population tended towards ramet growth rather than a density increase. A sudden drawdown of the ramet density also suggested an intense competition between ramets. In autumn, the ramet density increased but the average ramet biomass decreased, probably due to the growth of young ramets.

Based on the above evidence, we detected double effects of waves on intertidal vegetation growth at a salt marsh with artificial topography. First, the waves induced element enrichment through the sedimentation of biogenetic debris and mineral substances. Vegetation colonization and topography is an important factor for this accumulation process. Although artificial topography caused by the coastal development and direct wave exposure both have negative effects on the sexual reproduction of *S. alterniflora*, the mechanisms behind this phenomenon might be different. The inhibition caused by direct wave exposure was due to the biomass accumulation limit, while the inhibition caused by artificial topography was likely to be related to the transition of growth strategy triggered by the eutrophication. Moreover, ramet and population growth trait alterations were detected in response to element fractionations. An RDA based model suggests that Cu, Mn, N, Ni, S and As were critical in considering the relationship between the change in the sediment element composition and the alteration of the growth of *S. alterniflora*. These integrated effects of waves should be taken into consideration in the planning of coastal development.

## Methods

### Site setting

Investigation was performed at a semi-open abeyant seawall located at Rudong. Rudong is at the northeast edge of the Jiangsu Province, China (Fig. S1). The annual mean air temperature is 15 °C and precipitation is 1042 mm at Rudong. A majority (55–80%) of the precipitation occurs in the monsoon season from June to September. The bay has the feature of non-regular, semidiurnal tides. The seawall was constructed in 2010 according to information provided by local inhabitants and satellite images obtained using Google Earth. Due to the long-period that has passed since its construction, the direct impact of the construction on both the composition of the surficial sediment elements and growth of local vegetation could be reduced. *Spartina alterniflora* (Loisel.) was first introduced in the 1980s for seawall protection and silting promotion. After that, the continuous reclamation processes led to a loss of possible distribution zones for the native species. *S. alterniflora* was almost the only plant species present around the abeyant seawall, although a small amount of *Suaeda spp*. was fragmentarily distributed in the high marsh (Fig. S2).

As shown in Fig. S1, eight study sites with three plots in each site were established at the north-east edge of Rudong. Sites were divided into 3 groups. Three control (CL) sites were set at the mid and high marsh to represent the normal growth of *S. alterniflora* and two element accumulation (EA) sites were set before (EA1) and after (EA2) the seawall at approximately 150 meters apart to study the effect of artificial topography on the growth of *S. alterniflora*. It is well known that tidal flooding might cause growth differences and the halophytic population at the pioneer zone suffers plenty of stress from this. To take this tidal flooding derived effect into consideration, three direct wave exposure (WE) sites were set at the pioneer zones of the *S. alterniflora* population to investigate the stress on the vegetation growth by physical factors. This investigative strategy will help to reveal the actual roles of artificial topography and tidal transportation with the setting of CL and WE sites to clamp the effect related to the conventional zonation-drivers. Then, the effect of artificial topography might be identified in comparing the difference between EA and WE with CL as the background.

### Field survey

Data were collected in 2013 in the months of April, July, and November to estimate the general effects of artificial topography more comprehensively. The plant *Spartina alterniflora* sprout and seed at April and November, respectively and the aboveground biomass of *S. alterniflora* is highest at July^69^. The response of plant growth might be different at different stages, so the seasonal investigation is of great help on the description of complex response of plant. At each site, three 0.5 × 0.5 m^2^ plots were randomly selected with a spacing of more than 20 meters to avoid spatial autocorrelation. At each plot, all the aboveground parts of the vegetation with a height more than 5 cm were harvested sequentially to obtain the ramets. Every a certain number of ramets collected, the last ramet was selected and stored carefully. The number was determined by the ramet density in the plot. By this process, a subsample of 10–15 ramets was selected to avoid bias. Ten growth traits were collected throughout the survey. Ramet height, basal diameter, leaf number, and leaf thickness were measured *in situ* and the weights of the leaves and stems were acquired after the samples were rinsed well in a field laboratory. In total, the growth traits of 1354 ramets were measured. Surficial sediment samples at a depth of 0–10 cm were synchronously collected for element composition determination. After collection, the samples were immediately stored at 4 °C, transported to the laboratory and then stored at −20 °C prior to analysis.

### Element contents of sediment

Sediment and plant tissues were freeze-dried. Then, all samples were ground to a powder in a Wiley mill and passed through a 100-mesh sieve. The total carbon, nitrogen, and sulphur concentrations of the samples were directly analysed using an elemental analyser (Vario EL III, Elementar, Germany). Sediment samples were then treated with 1 M HCl to remove carbonate^70^. The unhydrolysed C concentrations obtained using the elemental analyser was categorized as the orgC. Total phosphorous concentrations of the sediments were analysed using a spectrophotometric method after acid digestion with a mixture of H_2_SO_4_/HClO_4_ for sediment^71^. The contents of Fe, Al, Mn, Cr, Ni, Cu, Zn, As, Cd, and Pb were determined using ICP-MS (Agilent 7700x, Agilent Technologies, USA) after pressurized acid digestion with a mixture of HNO_3_/HCLO_4_/HF. All chemicals used in the digestion were of reagent grade quality, and distilled deionized water was prepared with a Millipore water purification system (Barnstead, USA). Certified reference materials (GSD-12) were used for the assessment of validity and references between batches.

### Statistical analyses

R (version 3.3.1) was used for statistical analyses. Linear mixed models were performed using R package *lme4* ^72^ with brief Shapiro-Wilk tests of normality performed using R basic package *stats*. The models were automatically chosen with the Akaike information criterion as the criterion. Candidate fixed factors included group and season, while the site and the seasonal disturbance within site were considered as potential random factors. Analysis of variance (ANOVA) was performed using R basic package *stats* and package *car* (version 2.1–4) to analyse the spatio-temporal significant differences (p < 0.05) of both vegetation growth and element composition and the Tukey’s test was performed using R package *lsmeans* (version 2.25) for multiple comparisons. R package *merTools* (version 0.3.0) was then used to extract and visualize the fixed and random effects. Levene tests were performed to compare variances for growth traits between groups using R package *car*.

Plot-level ramet density, mean ramet height, mean ramet basal diameter and mean aboveground biomass were chosen as growth targets based on the ramet-level growth PCA results (Fig. S3) and cross-seasonal availability. Sediment element composition was chosen as the environment factor. The results from the foregoing Detrended Correspondence Analysis suggested a model derived from Redundancy Analysis (RDA) based on the linear model was more suitable for the depiction of the relationship between growth traits and element composition^73^. Due to the annoying distorted pattern of the gradient of the certain element caused by the growth trends between different seasons (Fig. S4), data of the growth traits needs to be detrended using Eq. (1) in advance to remove the ostensible seasonal growth trend:

1 $${E}_{i}=\,\mathrm{ln}(Growt{h}_{i}/\overline{Growt{h}_{{\rm{CL}}}})$$

where *E*_*i*_ means the growth promotion effect; *Growth*_*i*_ is the value of one certain growth trait at one plot; and $\overline{Growt{h}_{{\rm{CL}}}}$ means the mean of the same growth trait of all the plots in the CL group at the same season. The computing method for the pretreatment of the data was confirmed by comparing this algorithm to the (*index*-*background*)/*standard*_*deviation* algorithm (Fig. S5). Logarithmic transformation will achieve a more homogeneous sample distribution pattern. RDA was performed using R package *vegan* (version 2.4–1), and visualized using R packages *ggplot2* (version 2.1.0).

Data of the growth traits and element composition then underwent a further linear model fitting. The elements were selected based on the RDA result. To avoid interference from outliers (Fig. S6), robust linear regression using R package *MASS* (version 7.3–45) was chosen as the fitting algorithm, and visualization was performed using R package *ggplot2* (version 2.1.0).

### Data availability

The data reported in this article are partially available in the Supplementary information, and metadata can be acquired directly from the corresponding author C.Y. on request.

## Electronic supplementary material

Supplementary Information
