# Supplementary material for: Artificial topography changes the growth strategy of Spartina alterniflora, case study with wave exposure as a comparison
Source: Sci Rep. 2017 Nov 17;7:15768. doi: 10.1038/s41598-017-16122-w (PMC5693939; doi:10.1038/s41598-017-16122-w)
Supplement: Supplementary file 1 — Supplementary Information [file 41598_2017_16122_MOESM1_ESM.pdf]

Supplementary information for

Artificial topography changes the growth strategy of *Spartina alterniflora*, case study with wave exposure as a comparison

Hualong Hong[a], Minyue Dai[a], Haoliang Lu[a,b], Jingchun Liu[a],
Jie Zhang[c], Chaoqi Chen[d], Kang Xia[d,‡], Chongling Yan[a,b,†]

[a] Key Laboratory of the Ministry of Education for Coastal and Wetland Ecosystems, Xiamen University, Xiamen 361102, PR China

[b] State Key Laboratory of Marine Environmental Science, Xiamen University, Xiamen 361102, PR China

[c] Key Laboratory of Urban Environment and Health, Institute of Urban Environment, Chinese Academy of Sciences, Xiamen 361021, PR China

[d] Department of Crop and Soil Environmental Sciences, Virginia Tech, Blacksburg, VA 24061, USA

[†] Correspondence author: ycl@xmu.edu.cn

[‡] Correspondence author: kxia@vt.edu

Table of Content

Fig. S1 Map representing the sampling sites.

Fig. S2 Panorama of the landscape across the abeyant seawall.

Fig. S3 Relation between sediment element contents and distance from sea.

Fig. S4 Relation between plant growth and distance from sea.

Fig. S5 Plot of loading of components in PCA results for growth traits of *Spartina alterniflora*

Fig. S6 Distorted environmental factor on the projection plane caused by seasonal growth

Fig. S7 Comparison between two detrend algorithm.

Fig. S8 Comparison between results from robust linear regression (solid, red) and traditional linear regression (dashed, blue)

Fig. S9 Crabs activity traces

Fig. S10 Comparison of the allometry of *Spartina alterniflora* between different sites

Table S1 LMM fitting results for the sediment element composition

Table S2 Intragroup variation of element contents in each group

Table S3 Comparison between some element contents before and after the construction of the seawall in this study

Table S4 LMM fitting results for the growth traits of *Spartina alterniflora*

Table S5 Intragroup variation of vegetation growth traits in each group

Table S6 Multiple linear regression between element contents (N and Cu) and proxy indicating gather of plant litter (orgC) and accumulation of clay minerals (Al)

Table S7 ANOVA results for the fallen leaf count and seed setting rate in november

Table S8 Levene Test results of homogeneity of variances for the growth traits of *Spartina alterniflora*

Table S9 Outline of physiochemical parameters of the collected sediments

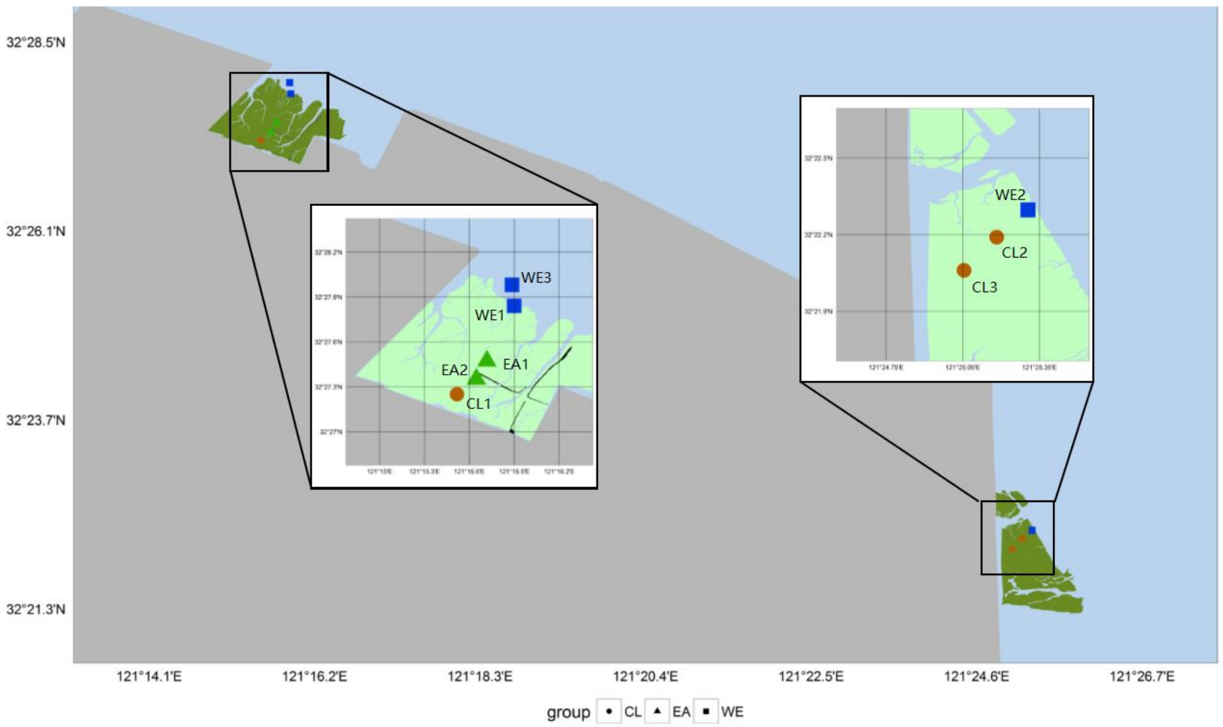

Fig. S1 Map representing the sampling sites.

Groups: CL, Control; EA, Element accumulation; WE, direct wave exposure.

The dark outline in the left submap represent the exist of the abeyant seawall.

Map was generated using R packages ggplot2 (version 2.1.0), ggmap (version 2.6.1) and maptools (version 0.8-39).

Map data source: Google, DigitalGlobe, TerraMetrics, Cnes/Spot Image and CNES / Astrium.

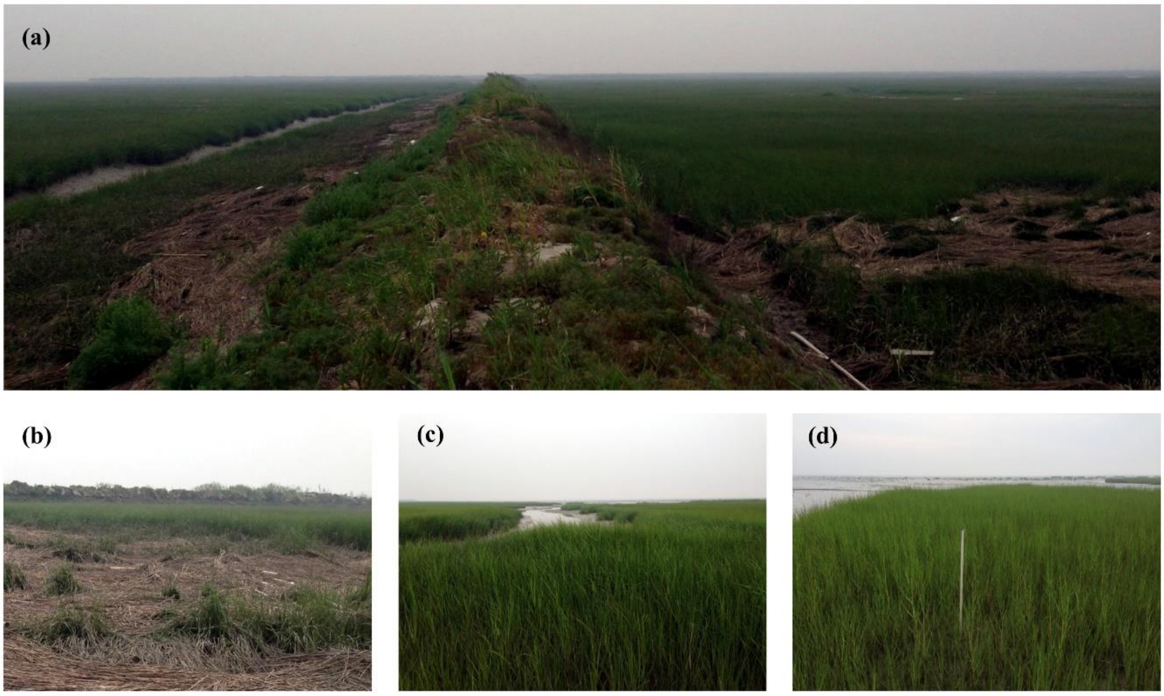

Fig. S2 Panorama of the landscape across the abeyant seawall.

Note (b) substantial accumulation of plant litter and (c, d) sites without the influence of the abeyant seawall. Photographs were taken by Hualong Hong.

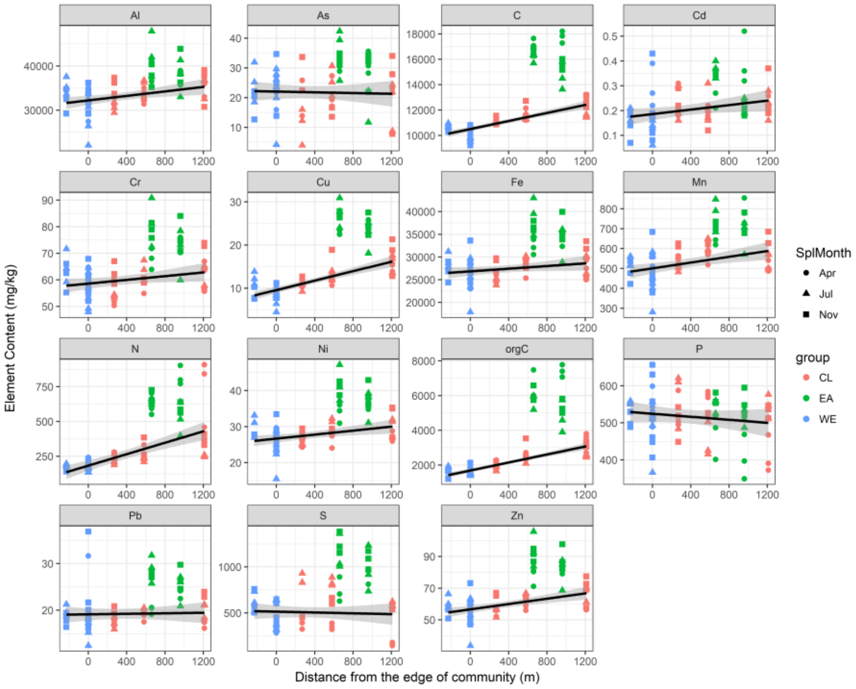

Fig. S3 Relation between sediment element contents and distance from sea.
Black line represent the relation between the element contents and the distance to the sea at the sites not affected by artificial topography (CL and WE).

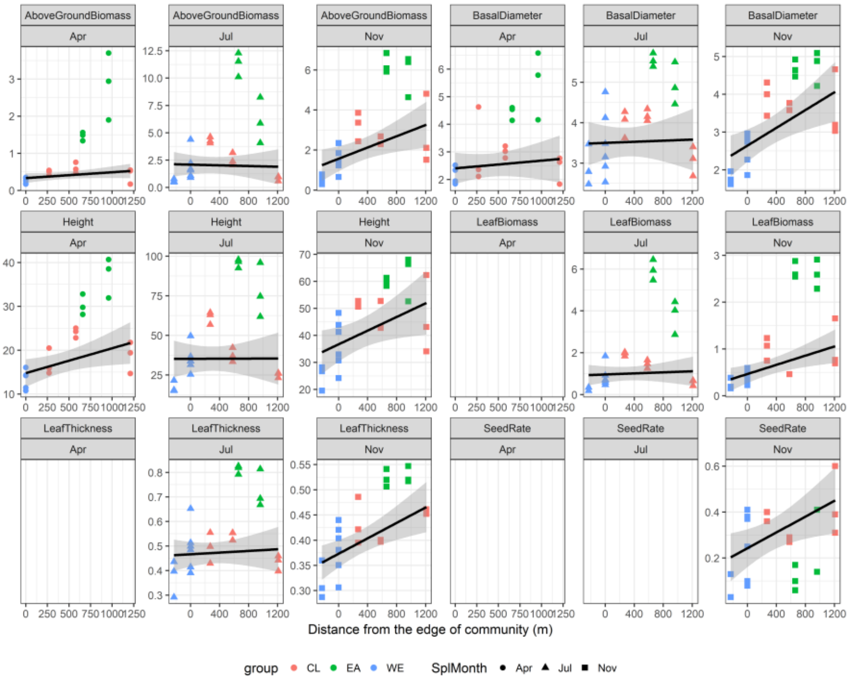

Fig. S4 Relation between plant growth and distance from sea.
Black line represent the relation between the growth traits and the distance to the sea at the sites not affected by artificial topography (CL and WE).

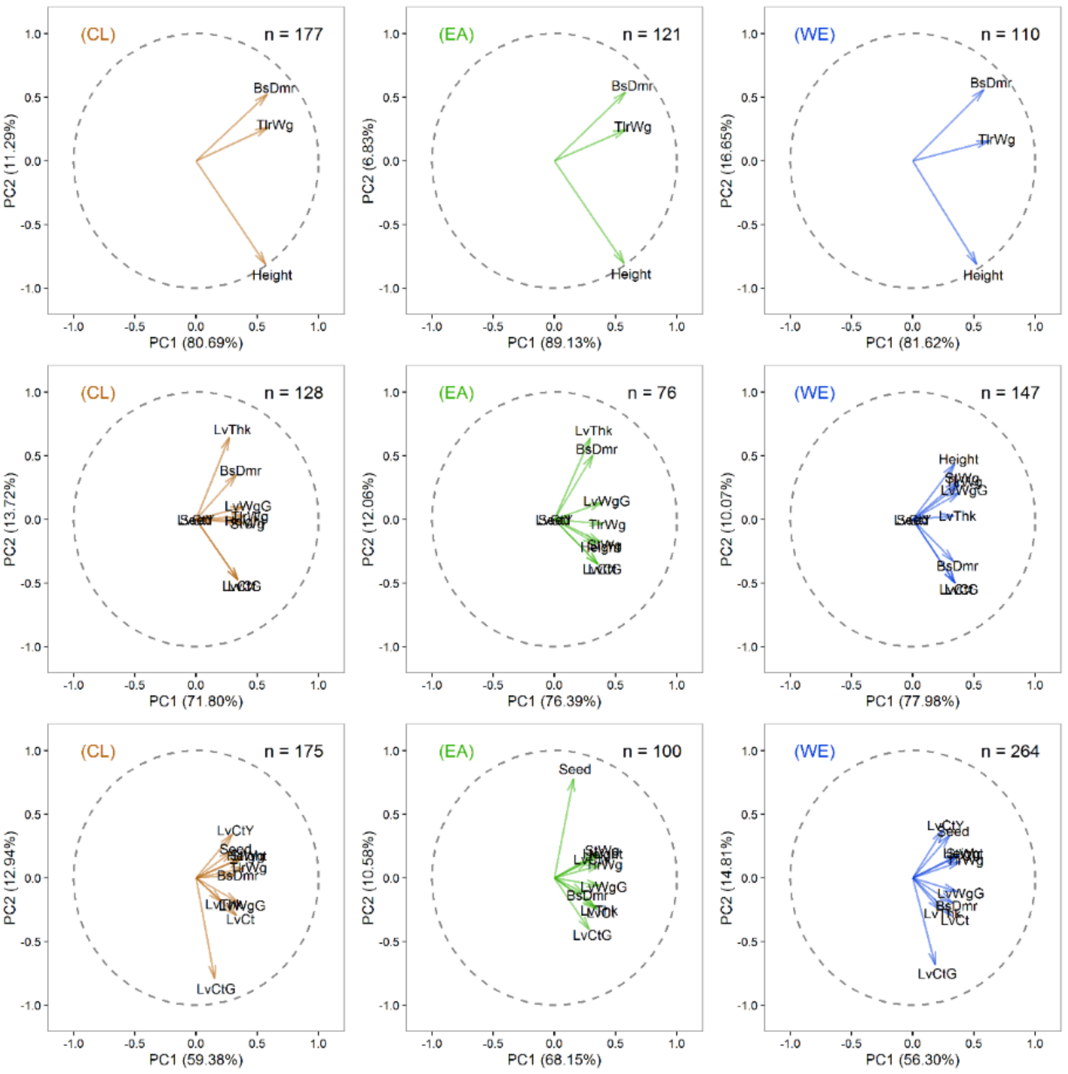

Fig. S5 Plot of loading of components in PCA results for growth traits of *Spartina alterniflora*

(Month: Top - Bottom) Apr-Jul-Nov; (group: Left – Right) CL-EA-WE

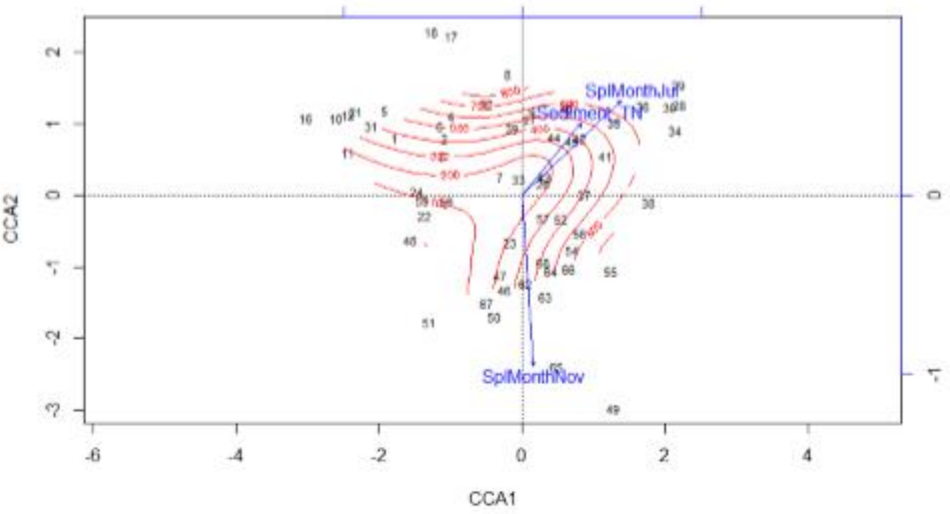

Fig. S6 Distorted environmental factor on the projection plane caused by seasonal growth

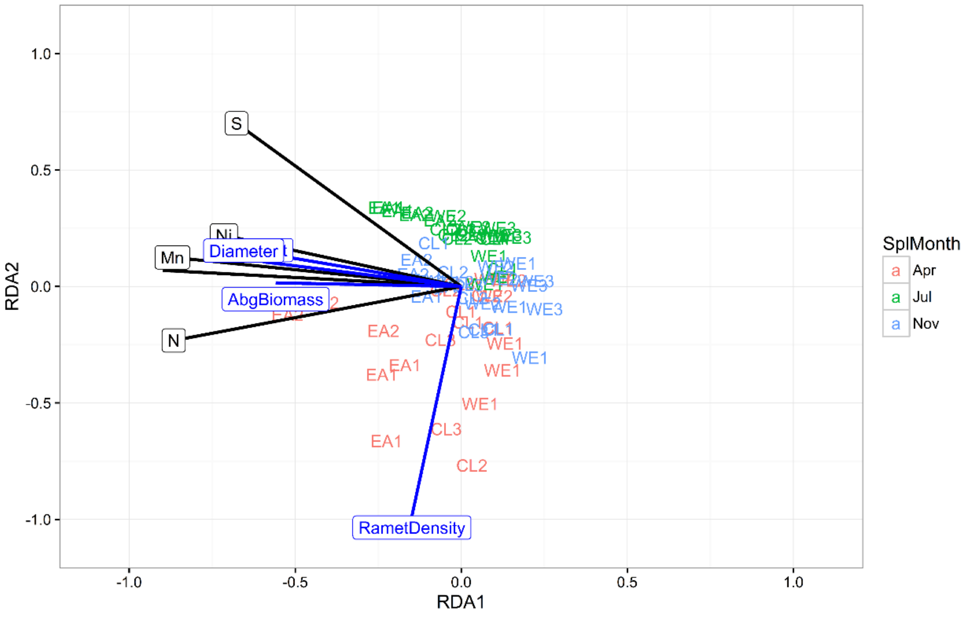

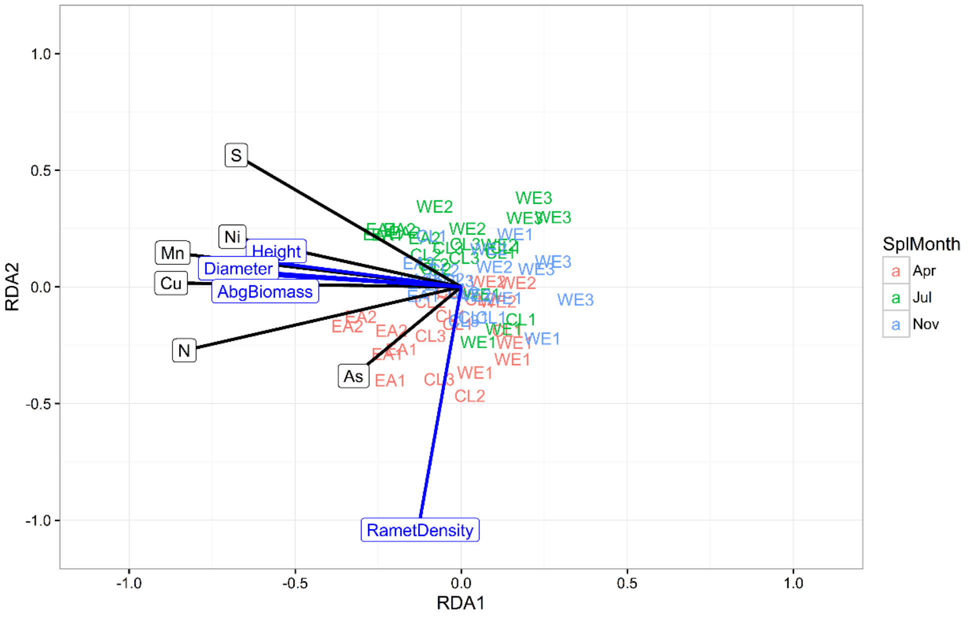

Fig. S7 Comparison between two detrend algorithm.

Top: $(\boldsymbol{index} - \boldsymbol{background})/\boldsymbol{standard\_deviation}$

Bottom: $\boldsymbol{E_i} = \ln\left(\boldsymbol{Grwoth_i}/\overline{\boldsymbol{Growth_{CL}}}\right)$.

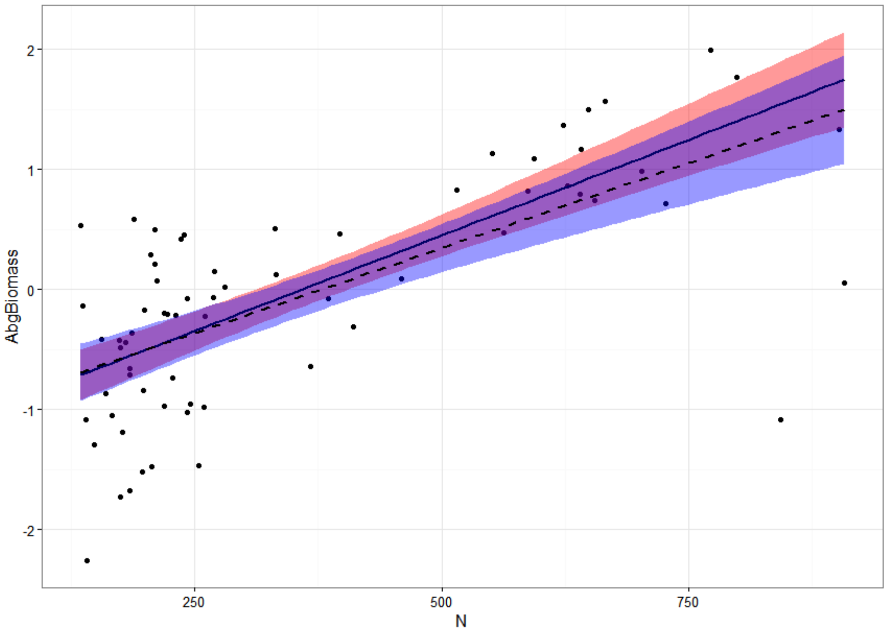

Fig. S8 Comparison between results from robust linear regression (solid, red) and traditional linear regression (dashed, blue)

note the outliers at the lower right quarter

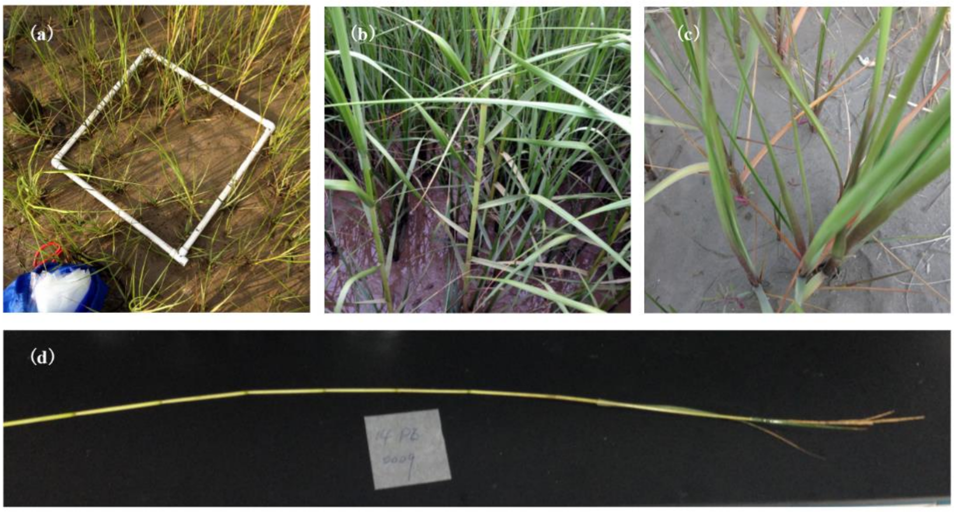

Fig. S9 Crabs activity traces

(a)caves distributed sporadically; (b-c) almost no gnawing traces at the leaf of *Spartina alterniflora*; (d) almost no gnawing traces at the stem of *Spartina alterniflora*

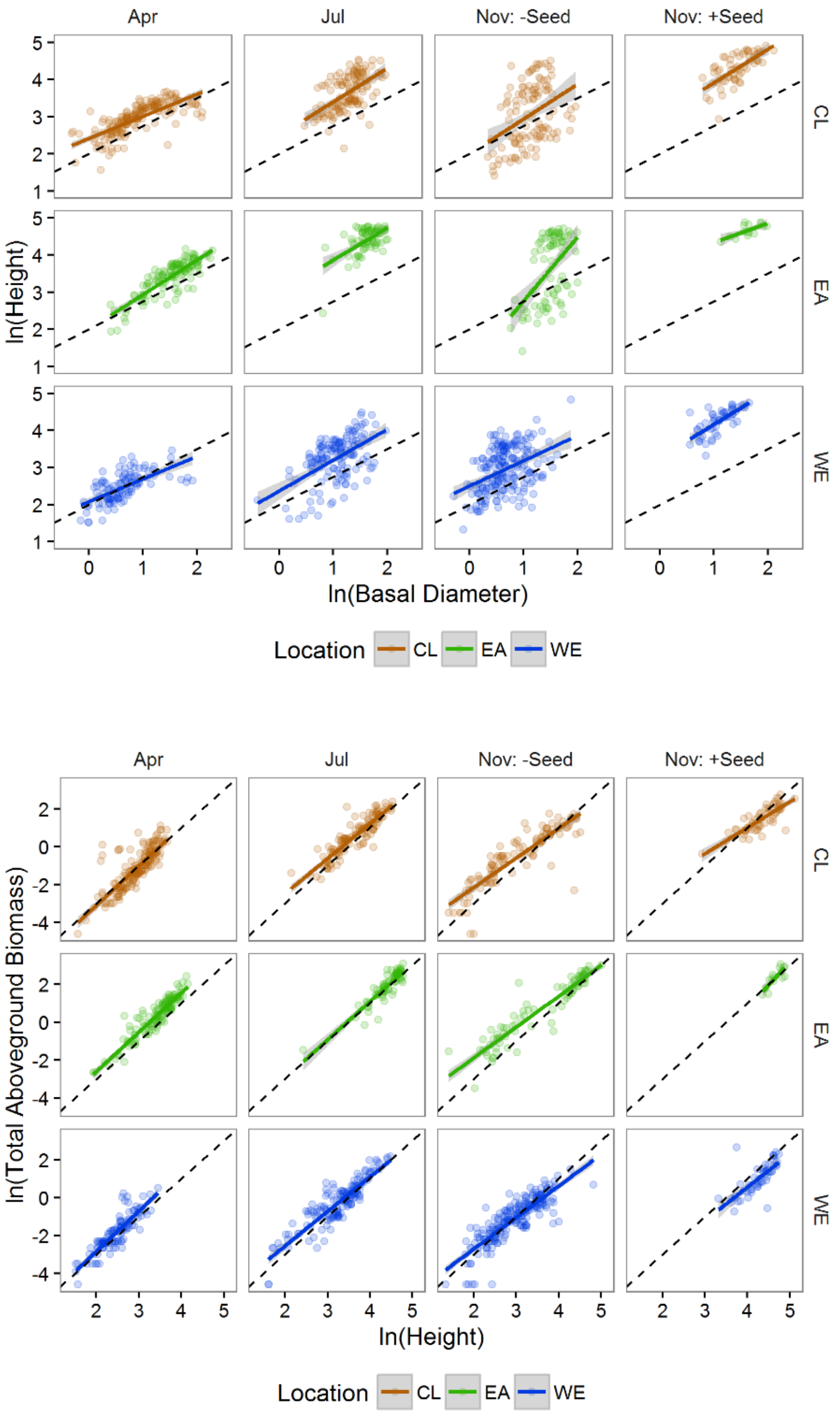

Fig. S10 Comparison of the allometry of *Spartina alterniflora* between different sites

Top: ramet height to basal diameter, the slope of dash line is 0.75.

Bottom: ramet aboveground biomass to height, the slope of dash line is 2.

Table S1 LMM fitting results for the sediment element composition

(a) Carbon

Model: C ~ group * SplMonth + (1 | SiteID) + (1 | SiteID:SplMonth)

	Df	Sum Sq	Mean Sq	F value	Pr(>F)	
group	2	27641447	13820724	83.76323	1.70E-36	***
SplMonth	2	1395557	697778.4	4.229025	0.014567	*
group:SplMonth	4	2218240	554559.9	3.36102	0.009298	**

PostHoc analysis:

Degrees-of-freedom method: satterthwaite, P value adjustment: tukey

group	SplMonth	lsmean	SE	df	lower.CL	upper.CL	
WE	Apr	10282.96	438.0913	12.81555	9335.139	11230.79	A
WE	Jul	10392.42	374.6847	10.46742	9562.598	11222.25	A
WE	Nov	10252.58	374.6847	10.46742	9422.755	11082.4	A
CL	Apr	11865.02	374.6847	10.46742	11035.2	12694.85	A
CL	Jul	11338.35	374.6847	10.46742	10508.53	12168.17	A
CL	Nov	11978.40	378.5144	10.88701	11144.24	12812.56	A
EA	Apr	17452.66	458.8932	10.46742	16436.34	18468.98	C
EA	Jul	15390.59	458.8932	10.46742	14374.26	16406.91	B
EA	Nov	16018.89	458.8932	10.46742	15002.57	17035.21	BC

***: p<0.001, **: 0.001<p<0.01, *: 0.01<p<0.05

(b) Organic carbon

Model: orgC ~ group * SplMonth + (1 | SiteID) + (1 | SiteID:SplMonth)

	Df	Sum Sq	Mean Sq	F value	Pr(>F)	
group	2	27507324	13753662	92.90831	2.57E-40	***
SplMonth	2	1425864	712932.1	4.815977	0.008099	**
group:SplMonth	4	1793148	448287.1	3.028255	0.01653	*

PostHoc analysis:

Degrees-of-freedom method: satterthwaite, P value adjustment: tukey

group	SplMonth	lsmean	SE	df	lower.CL	upper.CL	
WE	Apr	1600.802	338.5109	13.53803	872.4373	2329.167	A
WE	Jul	1597.12	283.0093	12.1155	981.1471	2213.093	A
WE	Nov	1588.366	283.0093	12.1155	972.3927	2204.338	A
CL	Apr	2665.05	283.0093	12.1155	2049.077	3281.023	A
CL	Jul	2194.49	283.0093	12.1155	1578.517	2810.463	A
CL	Nov	2704.194	287.461	12.84751	2082.423	3325.966	A
EA	Apr	6956.115	346.6141	12.1155	6201.705	7710.525	C
EA	Jul	5227.722	346.6141	12.1155	4473.312	5982.131	B
EA	Nov	5713.937	346.6141	12.1155	4959.527	6468.346	BC

***: p<0.001, **: 0.001<p<0.01, *: 0.01<p<0.05

(c) Nitrogen

Model: N ~ group + SplMonth + (1 | SiteID:SplMonth)

	Df	Sum Sq	Mean Sq	F value	Pr(>F)	
group	2	383223.9	191611.9	90.60546	2.59E-39	***
SplMonth	2	10980.99	5490.496	2.596232	0.074554	

PostHoc analysis:

Degrees-of-freedom method: satterthwaite, P value adjustment: tukey

group	SplMonth	lsmean	SE	df	lower.CL	upper.CL	
WE	Apr	219.8731	32.67386	16.89895	150.9058	288.8403	AB
WE	Jul	142.4519	29.62853	16.63419	79.83626	205.0675	A
WE	Nov	190.4661	29.65179	16.68311	127.8156	253.1165	AB
CL	Apr	318.8572	30.57991	18.04372	254.6223	383.0920	B
CL	Jul	241.4360	29.42084	16.78389	179.3025	303.5695	AB
CL	Nov	289.4502	29.61037	17.20561	227.0346	351.8658	AB
EA	Apr	680.4694	33.76726	16.81175	609.1659	751.7729	C
EA	Jul	603.0482	33.23773	16.64138	532.8074	673.2890	C
EA	Nov	651.0624	33.26538	16.69323	580.7802	721.3446	C

***: p<0.001, **: 0.001<p<0.01, *: 0.01<p<0.05

(d) Sulphur

Model: S ~ group + SplMonth + (1 | SiteID:SplMonth)

	Df	Sum Sq	Mean Sq	F value	Pr(>F)	
group	2	649517.3	324758.6	32.74805	1.10E-15	***
SplMonth	2	217919.3	108959.7	10.98728	1.69E-05	***

PostHoc analysis:

Degrees-of-freedom method: satterthwaite, P value adjustment: tukey

group	SplMonth	lsmean	SE	df	lower.CL	upper.CL	
WE	Apr	264.8538	66.03713	18.02124	126.1267	403.5810	AB
WE	Jul	574.5525	60.17341	18.01877	448.1423	700.9627	CD
WE	Nov	519.5505	60.22853	18.08021	393.0552	646.0457	CD
CL	Apr	348.6864	60.59507	18.04925	221.4058	475.9670	A C
CL	Jul	658.3851	59.63576	18.06407	533.1268	783.6433	D
CL	Nov	603.3830	60.06694	18.55277	477.4560	729.3100	B D
EA	Apr	833.1749	68.34645	18.01293	689.5917	976.7581	D
EA	Jul	1142.874	67.48101	18.00726	1001.105	1284.642	E
EA	Nov	1087.872	67.54653	18.07237	946.0022	1229.741	E

***: p<0.001, **: 0.001<p<0.01, *: 0.01<p<0.05

(e) Iron

Model: Fe ~ group + SplMonth + (1 | SiteID:SplMonth)

	Df	Sum Sq	Mean Sq	F value	Pr(>F)	
group	2	5.56E+08	2.78E+08	38.41473	1.07E-17	***

SplMonth	2	38406158	19203079	2.654901	0.070306	

PostHoc analysis:

Degrees-of-freedom method: satterthwaite, P value adjustment: tukey

group	SplMonth	lsmean	SE	df	lower.CL	upper.CL	
WE	Apr	25531.75	947.9198	18.01421	23540.35	27523.14	A
WE	Jul	26931.45	863.686	18.00789	25116.97	28745.93	A
WE	Nov	27845.31	866.0684	18.16524	26026.96	29663.67	A
CL	Apr	26507.52	870.5337	18.08596	24679.22	28335.82	A
CL	Jul	27907.22	857.1314	18.12389	26107.34	29707.1	A
CL	Nov	28821.09	875.629	19.37512	26990.77	30651.4	A
EA	Apr	34097.3	980.8235	17.99293	32036.61	36157.99	B
EA	Jul	35497	968.2389	17.97843	33462.63	37531.37	B
EA	Nov	36410.87	971.0721	18.14517	34371.89	38449.85	B

***: p<0.001, **: 0.001<p<0.01, *: 0.01<p<0.05

(f) Zinc

Model: Zn ~ group + SplMonth + (1 | SiteID)

	Df	Sum Sq	Mean Sq	F value	Pr(>F)	
group	2	4917.107	2458.554	47.15822	1.43E-21	***
SplMonth	2	320.4284	160.2142	3.073114	0.046277	*

PostHoc analysis:

Degrees-of-freedom method: satterthwaite, P value adjustment: tukey

group	SplMonth	lsmean	SE	df	lower.CL	upper.CL	
WE	Apr	52.96591	2.493244	11.83113	47.52498	58.40683	A
WE	Jul	56.32533	2.321197	9.657367	51.1284	61.52226	A
WE	Nov	58.39363	2.326984	9.748753	53.19061	63.59664	A
CL	Apr	59.57425	2.335421	9.844157	54.35942	64.78908	A
CL	Jul	62.93367	2.30884	9.518659	57.75378	68.11357	A
CL	Nov	65.00197	2.35533	10.21497	59.7689	70.23504	A
EA	Apr	82.77706	2.710046	8.043936	76.53362	89.0205	B
EA	Jul	86.13649	2.685177	7.819677	79.91952	92.35346	B
EA	Nov	88.20478	2.692174	7.892942	81.98193	94.42763	B

***: p<0.001, **: 0.001<p<0.01, *: 0.01<p<0.05

(g) Chromium

Model: Cr ~ group + SplMonth + (1 | SiteID)

	Df	Sum Sq	Mean Sq	F value	Pr(>F)	
group	2	1344.433	672.2167	19.18146	3.15E-09	***
SplMonth	2	221.0891	110.5445	3.154348	0.042666	*

PostHoc analysis:

Degrees-of-freedom method: satterthwaite, P value adjustment: tukey

group	SplMonth	lsmean	SE	df	lower.CL	upper.CL	
WE	Apr	56.48247	2.120383	10.48453	51.7874	61.17755	A
WE	Jul	58.89956	1.982271	8.563123	54.38024	63.41889	A
WE	Nov	61.02017	1.986827	8.63927	56.49689	65.54346	AB
CL	Apr	58.29895	1.993617	8.724391	53.76726	62.83063	A
CL	Jul	60.71603	1.97233	8.43959	56.20874	65.22333	AB
CL	Nov	62.83664	2.009117	9.02628	58.29372	67.37956	ABC
EA	Apr	72.36785	2.323396	7.221112	66.90781	77.82789	BCD
EA	Jul	74.78494	2.303557	7.025847	69.34195	80.22792	CD
EA	Nov	76.90555	2.309075	7.087684	71.45911	82.35198	D

***: p<0.001, **: 0.001<p<0.01, *: 0.01<p<0.05

(h) Nickel

Model: Ni ~ group + SplMonth + (1 | SiteID:SplMonth)

	Df	Sum Sq	Mean Sq	F value	Pr(>F)	
group	2	728.0124	364.0062	38.71838	7.58E-18	***
SplMonth	2	40.69235	20.34617	2.164168	0.114845	

PostHoc analysis:

Degrees-of-freedom method: satterthwaite, P value adjustment: tukey

group	SplMonth	lsmean	SE	df	lower.CL	upper.CL	
WE	Apr	24.98388	1.245535	18.0007	22.36711	27.60064	A
WE	Jul	26.67256	1.134881	17.99542	24.28822	29.0569	A
WE	Nov	27.72152	1.137365	18.12692	25.33321	30.10984	A
CL	Apr	27.04216	1.143555	18.06067	24.64021	29.4441	A
CL	Jul	28.73084	1.125796	18.09237	26.36649	31.09518	A
CL	Nov	29.7798	1.145132	19.13774	27.38418	32.17542	A
EA	Apr	36.57956	1.288869	17.98291	33.87156	39.28755	B
EA	Jul	38.26824	1.272399	17.9708	35.59472	40.94176	B
EA	Nov	39.3172	1.275353	18.11015	36.63895	41.99545	B

***: p<0.001, **: 0.001<p<0.01, *: 0.01<p<0.05

(i) Lead

Model: Pb ~ group + SplMonth + (1 | SiteID:SplMonth)

	Df	Sum Sq	Mean Sq	F value	Pr(>F)	
group	2	322.1963	161.0982	14.5123	3.98E-07	***
SplMonth	2	61.34749	30.67375	2.763201	0.063089	

PostHoc analysis:

Degrees-of-freedom method: satterthwaite, P value adjustment: tukey

group	SplMonth	lsmean	SE	df	lower.CL	upper.CL	
WE	Apr	17.87181	1.17401	18.0064	15.40537	20.33825	A
WE	Jul	19.1445	1.069685	18.00009	16.89718	21.39182	AB
WE	Nov	20.78985	1.072637	18.15721	18.53772	23.04198	ABC
CL	Apr	17.65636	1.078167	18.07806	15.39192	19.92081	A
CL	Jul	18.92906	1.061568	18.11593	16.69981	21.1583	AB
CL	Nov	20.57441	1.084487	19.36238	18.30742	22.84139	ABC
EA	Apr	23.92198	1.214761	17.98514	21.36971	26.47425	BCD
EA	Jul	25.19467	1.199175	17.97066	22.675	27.71434	CD
EA	Nov	26.84002	1.202685	18.13717	24.31464	29.3654	D

***: p<0.001, **: 0.001<p<0.01, *: 0.01<p<0.05

(j) Aluminium

Model: Al ~ group + (1 | SiteID:SplMonth)

	Df	Sum Sq	Mean Sq	F value	Pr(>F)	
group	2	3.43E+08	1.71E+08	19.10651	5.04E-09	***

PostHoc analysis:

Degrees-of-freedom method: satterthwaite, P value adjustment: tukey

group	lsmean	SE	df	lower.CL	upper.CL	
WE	31965.84	739.3329	19.88044	30423.02	33508.65	A
CL	34059.26	707.1259	20.76249	32587.68	35530.83	A
EA	38862.95	853.7081	19.88044	37081.46	40644.44	B

***: p<0.001, **: 0.001<p<0.01, *: 0.01<p<0.05

(k) Manganese

Model: Mn ~ group + (1 | SiteID:SplMonth)

	Df	Sum Sq	Mean Sq	F value	Pr(>F)	
group	2	225167.2	112583.6	33.97991	1.75E-15	***

PostHoc analysis:

Degrees-of-freedom method: satterthwaite, P value adjustment: tukey

group	lsmean	SE	df	lower.CL	upper.CL	
WE	478.5092	19.24252	19.89416	438.3563	518.662	A
CL	568.3681	18.30249	20.50114	530.2495	606.4866	B
EA	720.2067	22.21934	19.89416	673.8421	766.5712	C

***: p<0.001, **: 0.001<p<0.01, *: 0.01<p<0.05

(l) Copper

Model: Cu ~ group + (1 | SiteID:SplMonth)

	Df	Sum Sq	Mean Sq	F value	Pr(>F)	
group	2	725.4167	362.7083	94.29718	1.11E-41	***

PostHoc analysis:

Degrees-of-freedom method: satterthwaite, P value adjustment: tukey

group	lsmean	SE	df	lower.CL	upper.CL	
WE	9.0825	0.771708	19.75649	7.471473	10.69353	A
CL	13.6037	0.732415	20.2301	12.07702	15.13038	B
EA	25.00889	0.891091	19.75649	23.14864	26.86914	C

***: p<0.001, **: 0.001<p<0.01, *: 0.01<p<0.05

(m) Arsenic

Model: As ~ group + (1 | SiteID:SplMonth)

	Df	Sum Sq	Mean Sq	F value	Pr(>F)	
group	2	523.879	261.9395	6.152794	0.002128	**

PostHoc analysis:

Degrees-of-freedom method: satterthwaite, P value adjustment: tukey

group	lsmean	SE	df	lower.CL	upper.CL	
WE	22.83667	1.746769	19.58212	19.18798	26.48535	A
CL	20.83797	1.66787	20.38503	17.36306	24.31288	A
EA	29.80278	2.016995	19.58212	25.58964	34.01592	B

***: p<0.001, **: 0.001<p<0.01, *: 0.01<p<0.05

(n) Cadnium

Model: Cd ~ group + (1 | SiteID:SplMonth)

	Df	Sum Sq	Mean Sq	F value	Pr(>F)	
group	2	0.087688	0.043844	8.877839	0.000139	***

PostHoc analysis:

Degrees-of-freedom method: satterthwaite, P value adjustment: tukey

group	lsmean	SE	df	lower.CL	upper.CL	
WE	0.179583	0.019292	19.63152	0.139292	0.219875	A
CL	0.219961	0.018412	20.41211	0.181605	0.258318	A
EA	0.302778	0.022277	19.63152	0.256254	0.349302	B

***: p<0.001, **: 0.001<p<0.01, *: 0.01<p<0.05

(o) Phosphorus

Model: P ~ SplMonth + (1 | SiteID:SplMonth)

	Df	Sum Sq	Mean Sq	F value	Pr(>F)
SplMonth	2	3401.671	1700.836	0.870003	0.41895

Table S2 Intragroup variation of element contents in each group

Element	CL				EA			WE			
	**CL1**	**CL2**	**CL3**	**p**	**EA1**	**EA2**	**p**	**WE1**	**WE2**	**WE3**	**p**
Al	35400±900	32700±900	34000±700	0.077	39500±1300	38200±1100	0.431	31800±1400	31000±1000	33800±1100	0.317
As	21.9±2.88	19.6±2.97	21.3±2.08	0.814	33±1.72	26.6±2.57	0.052	25.9±0.748	20.5±2.88	21.8±2.65	0.194
C	12300±200	11100±100	11600±200	***	16600±200	16000±500	0.285	9800±100	10500±100	10600±100	***
Cd	0.241±0.0204	0.227±0.0193	0.191±0.0199	0.223	0.332±0.0193	0.273±0.037	0.178	0.201±0.0345	0.172±0.0296	0.158±0.0202	0.625
Cr	64.1±2.19	56.9±1.83	60.6±1.29	*	76±2.69	73.3±2.19	0.452	56.7±1.63	58.3±2.14	63.1±2.31	0.115
Cu	16.2±0.909	11.1±0.35	13.3±0.875	***	25.8±0.953	24.2±0.921	0.244	8.53±0.603	8.75±0.434	10.4±1	0.139
Fe	28800±1000	26100±700	28300±500	*	36000±1300	34600±1000	0.412	26100±1200	27000±1100	28000±900	0.561
Mn	561±21.7	544±16.2	602±15.8	0.098	718±23.3	723±26.6	0.886	412±18.6	528±26.8	505±20	**
N	453±83.5	225±10.6	271±20.8	*	643±17.8	646±51.9	0.960	199±8.8	166±9.68	171±8.81	*
Ni	30.5±1.14	26.3±0.55	28.7±0.885	**	38.8±1.62	37.3±1.08	0.429	25.4±1.47	26.3±1.15	29±1.02	0.187
orgC	3040±160	1960±70	2540±160	***	6090±210	5840±440	0.614	1600±40	1600±80	1600±120	0.997
P	496±23.9	534±20	515±23	0.468	527±18.2	490±25.4	0.256	490±14.4	545±28.7	530±12.4	0.176
Pb	19.6±0.9	17.9±0.503	19.5±0.342	0.138	25.6±1.49	25±0.869	0.753	19.9±2.25	19.3±1.68	18.8±0.676	0.922
S	443±70.6	562±77.2	603±80.1	0.318	1040±89.2	1000±54.7	0.693	415±43	444±51	617±43.8	*
Zn	67.1±2.36	57.6±1.75	62.5±1.28	**	87.5±3.33	83.9±2.56	0.402	54.6±3.11	56.5±2.71	58.2±2.05	0.685

Data presented as mean ± standard error. P value: * $0.01<p<0.05$; ** $0.001<p<0.01$; *** $p<0.001$.

Table S3 Comparison between some element contents before and after the construction of the seawall in this study

Element	2010 (before construction)		2013 (after construction)		Ratio (after/before)		T-test result	
	CL [1,2]	EA [1,2]	CL [1,3]	EA [1,3]	CL [4]	EA [4]	t	p [5]
As	5.90±0.63	8.63±1.34	20.9±1.52	29.8±1.69	3.54±0.26	3.45±0.20	0.286	0.77
Cd	0.156±0.013	0.12 [6]	0.221±0.012	0.303±0.022	1.42±0.08	2.52±0.18	-5.70	***
Cr	57.3±3.25	58.2±3.1	60.5±1.2	74.7±1.7	1.06±0.02	1.28±0.03	-6.32	***
Cu	8.36±1.31	11.2±1.1	13.5±0.6	25.0±0.7	1.62±0.07	2.23±0.06	-6.56	***
Ni	26.7±1.17	27.4±0.8	28.5±0.6	38.1±1.0	1.07±0.02	1.39±0.04	-7.62	***
Pb	7.04±0.44	7.51±0.50	19.0±0.4	25.3±0.8	2.70±0.06	3.37±0.12	-5.38	***
Zn	99.2±5.5	85.5±5.9	62.4±1.32	85.7±2.1	0.63±0.01	1.00±0.02	-13.4	***

Note:

1 units: mg/kg, data presented as mean ± standard error

2 data source.(In Chinese): Zhang, L., Du, Y., Wang, D., Gao, Sh. & Gao, W. Distribution Patterns and Pollution Assessments of Heavy Metals in the *Spartina alterniflora* Salt- Marsh Wetland of Rudong Jiangsu Province. *Environ. Sci*. **35**, 2401–2410 (2014)

3 data source: this study.

4 calculation: Ratio = $c_{after}/(\overline{c_{before}})$

5 ***: p<0.001, **: 0.001<p<0.01, *: 0.01<p<0.05

6 standard error not available

Table S4 LMM fitting results for the growth traits of *Spartina alterniflora*

(a) Ramet Density

Model: Density ~ SplMonth + (1 | SiteID)

	Df	Sum Sq	Mean Sq	F value	Pr(>F)	
SplMonth	2	20804.57	10402.28	11.34523	1.18E-05	***

PostHoc analysis:

Degrees-of-freedom method: satterthwaite, P value adjustment: tukey

SplMonth	lsmean	SE	df	lower.CL	upper.CL	
Apr	85.33188	9.138696	16.34507	65.9919	104.6719	A
Jul	72.375	8.73141	14.24028	53.67759	91.07241	A
Nov	113.7522	8.833809	14.84048	94.90573	132.5987	B

***: p<0.001, **: 0.001<p<0.01, *: 0.01<p<0.05

(b) Mean Ramet Height

Model: Height ~ group * SplMonth + (1 | SiteID:SplMonth)

	Df	Sum Sq	Mean Sq	F value	Pr(>F)	
group	2	1753.638	876.8189	21.10153	5.99E-11	***
SplMonth	2	1540.669	770.3347	18.53888	8.89E-09	***
group:SplMonth	4	470.8071	117.7018	2.832612	0.023091	*

PostHoc analysis:

Degrees-of-freedom method: satterthwaite, P value adjustment: tukey

group	SplMonth	lsmean	SE	df	lower.CL	upper.CL	
WE	Apr	12.94167	6.925171	13.99668	-1.91168	27.79501	A
WE	Jul	29.19	5.654378	13.99668	17.06229	41.31771	ABC
WE	Nov	32.88444	5.654378	13.99668	20.75674	45.01215	ABC
CL	Apr	19.91222	5.654378	13.99668	7.784517	32.03993	AB
CL	Jul	41.33889	5.654378	13.99668	29.21118	53.46659	ABC
CL	Nov	48.72227	5.718928	14.61323	36.5045	60.94004	BC
EA	Apr	33.645	6.925171	13.99668	18.79166	48.49834	ABC
EA	Jul	86.52667	6.925171	13.99668	71.67332	101.38	D
EA	Nov	61.05167	6.925171	13.99668	46.19832	75.90501	CD

***: p<0.001, **: 0.001<p<0.01, *: 0.01<p<0.05

(c) Mean Ramet Basal Diameter

Model: BasalDiameter ~ group * SplMonth + (1 | SiteID)

	Df	Sum Sq	Mean Sq	F value	Pr(>F)	
group	2	16.34193	8.170963	24.33577	7.73E-12	***
SplMonth	2	9.131438	4.565719	13.59819	1.24E-06	***
group:SplMonth	4	4.562681	1.14067	3.397285	0.008729	**

PostHoc analysis:

Degrees-of-freedom method: satterthwaite, P value adjustment: tukey

group	SplMonth	lsmean	SE	df	lower.CL	upper.CL	
WE	Apr	1.930162	0.308578	16.29433	1.276963	2.58336	A
WE	Jul	3.3	0.265689	10.61893	2.712653	3.887347	BC
WE	Nov	2.301111	0.265689	10.61893	1.713764	2.888458	A
CL	Apr	2.815556	0.265689	10.61893	2.228208	3.402903	AB
CL	Jul	3.744444	0.265689	10.61893	3.157097	4.331792	CD
CL	Nov	3.764845	0.274873	11.99263	3.165908	4.363782	CD
EA	Apr	4.966667	0.325401	10.61893	4.247316	5.686017	D
EA	Jul	5.241667	0.325401	10.61893	4.522316	5.961017	D
EA	Nov	4.703333	0.325401	10.61893	3.983983	5.422684	CD

***: $p<0.001$, **: $0.001<p<0.01$, *: $0.01<p<0.05$

(d) Mean Ramet Aboveground Biomass

Model: AboveGroundBiomass ~ group * SplMonth + (1 | SiteID:SplMonth)

	Df	Sum Sq	Mean Sq	F value	Pr(>F)	
group	2	31.07733	15.53866	22.65803	2.90E-11	***
SplMonth	2	14.75784	7.378918	10.75973	2.12E-05	***
group:SplMonth	4	7.25685	1.814212	2.645432	0.03169	*

PostHoc analysis:

Degrees-of-freedom method: satterthwaite, P value adjustment: tukey

group	SplMonth	lsmean	SE	df	lower.CL	upper.CL	
WE	Apr	0.246667	0.925724	13.98969	-1.73895	2.232283	A
WE	Jul	1.52	0.75585	13.98969	-0.10125	3.141249	A
WE	Nov	1.166667	0.75585	13.98969	-0.45458	2.787916	A
CL	Apr	0.501111	0.75585	13.98969	-1.12014	2.12236	A
CL	Jul	2.564444	0.75585	13.98969	0.943195	4.185694	AB
CL	Nov	2.849719	0.763851	14.56282	1.217341	4.482096	AB
EA	Apr	2.155	0.925724	13.98969	0.169383	4.140617	AB
EA	Jul	8.686667	0.925724	13.98969	6.70105	10.67228	C
EA	Nov	6.068333	0.925724	13.98969	4.082717	8.05395	BC

***: $p<0.001$, **: $0.001<p<0.01$, *: $0.01<p<0.05$

(e) Mean Ramet Leaf Thickness

Model: LeafThickness ~ group * SplMonth + (1 | SiteID)

	Df	Sum Sq	Mean Sq	F value	Pr(>F)	
group	2	0.086422	0.043211	15.62943	1.44E-07	***
SplMonth	1	0.16152	0.16152	58.42224	2.11E-14	***
group:SplMonth	2	0.066863	0.033431	12.09218	5.60E-06	***

PostHoc analysis:

Degrees-of-freedom method: satterthwaite, P value adjustment: tukey

group	SplMonth	lsmean	SE	df	lower.CL	upper.CL	
WE	Jul	0.453171	0.030307	7.15215	0.381815	0.524528	B
WE	Nov	0.361521	0.030307	7.15215	0.290165	0.432877	A
CL	Jul	0.490271	0.030307	7.15215	0.418915	0.561628	AB
CL	Nov	0.435012	0.031003	7.781017	0.363168	0.506856	AB
EA	Jul	0.768724	0.037118	7.15215	0.68133	0.856117	C
EA	Nov	0.52532	0.037118	7.15215	0.437927	0.612714	AB

***: p<0.001, **: 0.001<p<0.01, *: 0.01<p<0.05

(f) Mean Green Leaf Count per Ramet

Model: GreenLeafCount ~ group + SplMonth + (1 | SiteID:SplMonth)

	Df	Sum Sq	Mean Sq	F value	Pr(>F)	
group	2	1.187078	0.593539	2.532945	0.079296	
SplMonth	1	1.806127	1.806127	7.707701	0.005499	**

PostHoc analysis:

Degrees-of-freedom method: satterthwaite, P value adjustment: tukey

group	SplMonth	lsmean	SE	df	lower.CL	upper.CL	
WE	Jul	4.207285	0.294299	11.9282	3.565635	4.848935	AB
WE	Nov	3.352046	0.294299	11.9282	2.710395	3.993696	A
CL	Jul	4.536344	0.294725	11.99233	3.894147	5.17854	AB
CL	Nov	3.681104	0.297271	12.37874	3.035598	4.326611	AB
EA	Jul	5.099346	0.343592	11.91858	4.350156	5.848537	B
EA	Nov	4.244107	0.343592	11.91858	3.494917	4.993298	AB

***: p<0.001, **: 0.001<p<0.01, *: 0.01<p<0.05

(g) Mean Ramet Leaf Biomass

Model: LeafBiomass ~ group * SplMonth + (1 | SiteID:SplMonth)

	Df	Sum Sq	Mean Sq	F value	Pr(>F)	
group	2	8.654983	4.327492	34.50426	1.01E-15	***
SplMonth	1	0.948557	0.948557	7.563104	0.005958	**
group:SplMonth	2	0.809507	0.404753	3.227207	0.039668	*

PostHoc analysis:

Degrees-of-freedom method: satterthwaite, P value adjustment: tukey

group	SplMonth	lsmean	SE	df	lower.CL	upper.CL	
WE	Jul	0.67826	0.360899	9.963714	-0.12627	1.482791	AB
WE	Nov	0.358914	0.360899	9.963714	-0.44562	1.163445	A
CL	Jul	1.33102	0.360899	9.963714	0.526488	2.135551	AB
CL	Nov	0.844304	0.363993	10.29667	0.036433	1.652175	AB
EA	Jul	4.86278	0.44201	9.963714	3.877435	5.848126	C
EA	Nov	2.632982	0.44201	9.963714	1.647637	3.618328	B

***: p<0.001, **: 0.001<p<0.01, *: 0.01<p<0.05

(h) Mean Ramet Stem Biomass

Model: StemBiomass ~ group + SplMonth + (1 | SiteID:SplMonth)

	Df	Sum Sq	Mean Sq	F value	Pr(>F)	
group	2	10.38301	5.191503	12.41813	4.05E-06	***
SplMonth	1	0.067822	0.067822	0.162232	0.68711	

PostHoc analysis:

Degrees-of-freedom method: satterthwaite, P value adjustment: tukey

group	SplMonth	lsmean	SE	df	lower.CL	upper.CL	
WE	Jul	0.734961	0.422368	12.0088	-0.18523	1.655147	A
WE	Nov	0.912985	0.422368	12.0088	-0.0072	1.833171	A
CL	Jul	1.528361	0.422904	12.06572	0.607489	2.449233	AB
CL	Nov	1.706385	0.426102	12.4085	0.781367	2.631404	A C
EA	Jul	3.541241	0.493126	12.00027	2.466814	4.615668	CD
EA	Nov	3.719265	0.493126	12.00027	2.644838	4.793692	B D

***: p<0.001, **: 0.001<p<0.01, *: 0.01<p<0.05

(i) Mean Fallen Leaf Count per Ramet

Model: FallenLeafCount ~ group + (1 | SiteID)

	Df	Sum Sq	Mean Sq	F value	Pr(>F)	
group	2	1.356187	0.678094	4.59388	0.010114	*

PostHoc analysis:

Degrees-of-freedom method: satterthwaite, P value adjustment: tukey

group	lsmean	SE	df	lower.CL	upper.CL	
WE	1.224296	0.158589	4.053449	0.786263	1.662328	A
CL	1.871991	0.165517	4.687105	1.43783	2.306152	A
EA	1.286887	0.194231	4.053449	0.750409	1.823365	A

***: p<0.001, **: 0.001<p<0.01, *: 0.01<p<0.05

(j) Mean Seed Setting Rate

Model: SeedRate ~ group + (1 | SiteID)

	Df	Sum Sq	Mean Sq	F value	Pr(>F)	
group	2	0.042741	0.02137	3.352472	0.034998	*

PostHoc analysis:

Degrees-of-freedom method: satterthwaite, P value adjustment: tukey

group	lsmean	SE	df	lower.CL	upper.CL	
WE	0.194444	0.069637	4.927607	0.014642	0.374247	A
CL	0.368678	0.070441	5.146897	0.189148	0.548209	A
EA	0.090705	0.086777	5.26546	-0.12902	0.310432	A

***: p<0.001, **: 0.001<p<0.01, *: 0.01<p<0.05

Table S5 Intragroup variation of vegetation growth traits in each group

Trait	Month	CL				EA			WE			
		CL1	CL2	CL3	p	EA1	EA2	p	WE1	WE2	WE3	p
Aboveground Biomass	Apr	0.42±0.12	0.49±0.04	0.60±0.09	0.401	1.46±0.06	2.85±0.52	0.058	0.24±0.06	0.25±0.05	NA	0.862
	Jul	0.85±0.13	4.31±0.17	2.54±0.33	***	11.3±0.63	6.06±1.21	*	1.39±0.24	2.57±0.97	0.61±0.09	0.132
	Nov	2.81±1.01	3.22±0.42	2.49±0.19	0.802	6.28±0.28	5.86±0.61	0.563	1.09±0.22	1.87±0.27	0.53±0.14	*
Basal Diameter	Apr	2.41±0.30	3.03±0.80	3.00±0.12	0.631	4.43±0.14	5.51±0.71	0.211	2.11±0.20	2.04±0.15	NA	0.788
	Jul	3.06±0.21	3.99±0.20	4.18±0.09	**	5.54±0.10	4.94±0.30	0.130	2.87±0.181	4.12±0.37	2.91±0.29	*
	Nov	3.63±0.52	3.91±0.26	3.67±0.10	0.85	4.68±0.13	4.73±0.26	0.864	2.31±0.275	2.82±0.07	1.77±0.10	*
Height	Apr	18.6±2.1	17.1±1.7	24.0±0.6	*	30.2±1.4	37.0±2.6	0.084	12±1.17	13.9±1.35	NA	0.333
	Jul	25.1±1.0	61.4±2.3	37.5±2.6	***	95.6±1.6	77.5±9.9	0.146	33.9±1.5	36.3±7.1	17.4±2.1	*
	Nov	46.5±8.3	51.8±0.6	47.8±5.0	0.796	59.7±0.9	62.4±4.9	0.623	29.3±2.63	44.5±2.05	24.8±2.7	**
Leaf Biomass	Jul	0.59±0.08	1.95±0.06	1.46±0.12	***	5.95±0.28	3.77±0.47	*	0.62±0.08	1.12±0.38	0.30±0.05	0.103
	Nov	1.04±0.31	1.02±0.14	0.46±0.0004	0.267	2.67±0.10	2.59±0.18	0.731	0.36±0.08	0.48±0.08	0.24±0.07	0.171
Leaf Thickness	Jul	0.43±0.02	0.49±0.03	0.54±0.01	*	0.81±0.01	0.72±0.04	0.131	0.43±0.03	0.555±0.05	0.38±0.04	0.051
	Nov	0.46±0.003	0.43±0.02	0.40±0.001	0.207	0.52±0.01	0.53±0.01	0.713	0.35±0.03	0.41±0.02	0.32±0.02	0.063
Seed Setting Rate	Nov	0.43±0.08	0.38±0.01	0.28±0.01	0.299	0.11±0.03	0.28±0.14	0.227	0.14±0.05	0.39±0.01	0.08±0.05	**

Data presented as mean ± standard error. P value: * $0.01<p<0.05$; ** $0.001<p<0.01$; *** $p<0.001$.

Table S6 Multiple linear regression between element contents (N and Cu) and proxy indicating gather of plant litter (orgC) and accumulation of clay minerals (Al)

	Intercept(mg/kg)		orgC(mg/kg)		Al(mg/kg)	
	Estimate±SE	p	Estimate±SE	p	Estimate±SE	p
N(mg/kg)	-4±115	0.974	0.105±0.008	***	9.9e-4±3.75e-3	0.792
Cu(mg/kg)	12.9±2.0	***	2.56e-3±1.5e-4	***	5.78e-4±6.6e-5	***

***: p<0.001, **: 0.001<p<0.01, *: 0.01<p<0.05

Table S7 ANOVA results for the fallen leaf count and seed setting rate in november

(a) Mean Seed Setting Rate

Model: SeedRate ~ SiteID

	Df	Sum Sq	Mean Sq	F value	Pr(>F)	
SiteID	7	0.48539	0.069342	10.843	0.000104	***
Residuals	14	0.08953	0.006395			

PostHoc analysis:

Degrees-of-freedom method: satterthwaite, P value adjustment: tukey

SiteID	lsmean	SE	df	lower.CL	upper.CL	
WE1	0.143333	0.046171	14	0.044307	0.24236	A
WE2	0.386667	0.046171	14	0.28764	0.485693	B
WE3	0.053333	0.046171	14	-0.04569	0.15236	A
CL1	0.433333	0.046171	14	0.334307	0.53236	B
CL2	0.386667	0.046171	14	0.28764	0.485693	B
CL3	0.28	0.056547	14	0.158718	0.401282	A B
EA1	0.11	0.046171	14	0.010973	0.209027	A
EA2	0.07	0.056547	14	-0.05128	0.191282	A

***: p<0.001, **: 0.001<p<0.01, *: 0.01<p<0.05

(b) Mean Fallen Leaf Count per Ramet

Model: FallenLeafCount ~ SiteID

	Df	Sum Sq	Mean Sq	F value	Pr(>F)	
SiteID	7	3.0922	0.44174	3.0653	0.03253	*
Residuals	15	2.1616	0.14411			

PostHoc analysis:

Degrees-of-freedom method: satterthwaite, P value adjustment: tukey

SiteID	lsmean	SE	df	lower.CL	upper.CL	
WE1	1.18939	0.219171	15	0.722238	1.656542	A
WE2	1.18385	0.219171	15	0.716697	1.651002	A
WE3	1.299647	0.219171	15	0.832495	1.7668	A
CL1	1.974704	0.219171	15	1.507551	2.441856	A
CL2	1.417677	0.219171	15	0.950525	1.884829	A
CL3	2.338235	0.268429	15	1.766093	2.910378	A
EA1	1.201225	0.219171	15	0.734073	1.668378	A
EA2	1.372549	0.219171	15	0.905397	1.839701	A

***: p<0.001, **: 0.001<p<0.01, *: 0.01<p<0.05

Table S8 Levene Test results of homogeneity of variances for the growth traits of *Spartina alterniflora*

(a) Mean Ramet Aboveground Biomass

group	SplMonth	mean	se	CV	Fvalue	Pr	
WE	Apr	0.246667	0.079162	0.320928	0.4993	0.4923	
WE	Jul	1.52	1.21958	0.802355	1.6118	0.2224	
WE	Nov	1.166667	0.667383	0.572043	1.908	0.1874	
CL	Apr	0.501111	0.156321	0.311948			
CL	Jul	2.564444	1.536572	0.599183			
CL	Nov	2.88625	1.064867	0.368945			
EA	Apr	2.155	0.951709	0.441628	21.094	0.000504	***
EA	Jul	8.686667	3.241368	0.373143	5.4927	0.03564	*
EA	Nov	6.068333	0.772487	0.127298	1.1858	0.2976	

***: p<0.001, **: 0.001<p<0.01, *: 0.01<p<0.05

(b) Density

group	SplMonth	mean	se	CV	Fvalue	Pr	
WE	Apr	71.66667	32.70882	0.456402	0.0936	0.7645	
WE	Jul	75.55556	54.0234	0.715016	9.0227	0.008413	**
WE	Nov	99.44444	36.52092	0.367249	0.0113	0.9169	
CL	Apr	85.33333	39.42715	0.462037			
CL	Jul	75.66667	21.0238	0.277848			
CL	Nov	125	34.35529	0.274842			
EA	Apr	109.8333	27.98154	0.254764	0.8316	0.3784	
EA	Jul	62.66667	2.33809	0.03731	4.7581	0.04812	*
EA	Nov	119.3333	13.30664	0.111508	5.9855	0.03079	*

***: p<0.001, **: 0.001<p<0.01, *: 0.01<p<0.05

Table S9 Outline of physiochemical parameters of the collected sediments

group	pH(1:5)	Carbonate (as TIC, g/kg)	Sand(%)	Silt(%)	Clay(%)
CL	6.77±0.04	9.21±0.05	0.3±0.2	79.7±2.1	20.0±2.3
CL(range)	6.10-6.99	8.49-9.81	0-0.8	77.0-83.8	15.4-23.0
EA	6.73±0.03	10.3±0.08	0.7±0.4	80.8±2.7	18.5±2.8
EA(range)	6.46-6.89	9.74-11.1	0.2-1.4	76.6-85.8	13.6-23.3
WE	6.78±0.04	8.69±0.09	0.4±0.2	83.1±0.9	16.5±1.1
WE(range)	6.14-6.98	7.66-9.37	0-0.8	82.1-84.9	14.3-17.6
